# In vivo confocal microscopy evaluation of infiltrated immune cells in corneal stroma treated with cell therapy in advanced keratoconus

**DOI:** 10.1186/s12348-024-00385-2

**Published:** 2024-01-26

**Authors:** Mona El Zarif, Karim Abdul Jawad, Jorge L. Alió, Nehman Makdissy, María P. De Miguel

**Affiliations:** 1 Optica General, Saida, Lebanon; 2 Division of Ophthalmology, Universidad Miguel Hernández, Alicante, Spain; 3 Doctoral School of Sciences and Technology, Lebanese University, Hadath, Lebanon; 4 Cornea, Cataract and Refractive Surgery Unit, Vissum (Miranza Group), Alicante, Spain; 5 Genomic Surveillance and Biotherapy GSBT, Faculty of Sciences, Lebanese University, RasMaska, Lebanon; 6 Cell Engineering Laboratory, IdiPAZ, La Paz Hospital Health Research Institute, Madrid, Spain

**Keywords:** Keratoconus, Immune cells, Cell therapy, Adipose-derived adult stem cells, Corneal laminas, Cell infiltration, In vivo confocal microscopy, Immune response, Tissue repair, Clinical trial

## Abstract

**Purpose:**

This study investigates immune cell (ICs) infiltration in advanced keratoconus patients undergoing autologous adipose-derived adult stem cell (ADASC) therapy with recellularized human donor corneal laminas (CL).

**Methods:**

A prospective clinical trial included fourteen patients divided into three groups: G-1, ADASCs; G-2, decellularized CL (dCL); and G-3, dCL recellularized with ADASCs (ADASCs-rCL). Infiltrated ICs were assessed using in vivo confocal microscopy (IVCM) at 1,3,6, and12 months post-transplant.

**Results:**

Infiltrated ICs, encompassing granulocytes and agranulocytes, were observed across all groups, categorized by luminosity, structure, and area. Stromal ICs infiltration ranged from 1.19% to 6.62%, with a consistent increase in group-related cell density (*F* = 10.68, *P* < .0001), independent of post-op time (*F* = 0.77, *P* = 0.511); the most substantial variations were observed in G-3 at 6 and 12 months (2.0 and 1.87-fold, respectively). Similarly, significant size increases were more group-dependent (*F* = 5.76, *P* < .005) rather than time-dependent (*F* = 2.84, *P* < .05); G-3 exhibited significant increases at 6 and 12 months (3.70-fold and 2.52-fold, respectively). A lamina-induced shift in IC size occurred (*F* = 110.23, *P* < .0001), primarily with 50–100 μm^2^ sizes and up to larger cells > 300μm^2^, presumably macrophages, notably in G-3, indicating a potential role in tissue repair and remodeling, explaining reductions in cells remnants < 50μm^2^.

**Conclusions:**

ADASCs-rCL therapy may lead to increased IC infiltration compared to ADASCs alone, impacting cell distribution and size due to the presence of the lamina. The findings reveal intricate immune patterns shaped by the corneal microenvironment and highlight the importance of understanding immune responses for the development of future therapeutic strategies.

## Introduction

Over the past decade, cellular therapy targeting the corneal stroma has emerged as a topic of significant interest. Mesenchymal stem cells, particularly adipose-derived adult stem cells (ADASCs), have shown remarkable potential for in vivo and in vitro differentiation into functional adult keratocytes [1]*.*

Noteworthy reports, including studies from our research group [2–4], have demonstrated the survival and differentiation of these cells into adult human keratocytes, contributing to collagen production within the host stroma [2–5] and enhancing corneal transparency in conditions like corneal dystrophies [6]. In a pioneering clinical intervention, our group conducted a prospective randomized study involving cases of advanced keratoconus, where patients received treatment via the implantation of autologous ADASCs alone or in combination with decellularized/recellularized human corneal stromal laminas (dCL/ADASCs-rCL). Summarizing a follow-up period spanning 36 months [7–11], our findings showcased notable improvement in visual acuity across all patients, with no clinically relevant inflammatory responses observed.

Our comprehensive confocal study investigating the cellular landscape of the intervened corneas revealed scattered infiltration of inflammatory cells. Traditionally, the corneal microenvironment has been characterized as anti-inflammatory and immunosuppressive. Yet instances of ocular infections prompt the recruitment of T cells into the tissue, potentially leading to immunopathological effects such as stromal keratitis [12]. CD4 + T cells orchestrate various aspects of disease during the clinical phase of HSV infection, while CD8 + T cells also infiltrate the cornea [13]. Recent research employing intravital 2-photon microscopy in mice has unveiled the presence of T lymphocytes on the corneal surface, showcasing the formation of tissue-resident memory T cells in response to ocular viral infections [14].

Previous studies have also documented the presence of such cell types in the corneal stroma of patients with keratitis fugax hereditaria, an autosomal dominant cryopyrin-associated periodic keratitis. During acute episodes, hyperreflective cellular structures indicative of inflammatory cells were observed transiently within the anterior to middle layers of the corneal stroma [15]. Instances of “microdots” were reported in the central cornea, with their prevalence increasing from preoperative (50%) to postoperative (90.9%) stages in patients undergoing femtosecond laser-assisted keratoplasty [16]. These corneal microdots, initially described in the context of long-term contact lens use [17], have also been identified following LASIK, DSAEK, and PK procedures [18–20]. Histological and ultrastructural investigations of these particles reveal a combination of intracellular and extracellular debris, encompassing degenerating cellular materials and inflammatory cells [21].

Furthermore, the presence of leukocytes has been noted in the corneal epithelium of aqueous tear-deficient dry eye conditions. While leukocyte density within the normal corneal epithelium remains low, it escalates in the central corneal epithelium in the case of non-Sjögren’s syndrome, and both the central and peripheral corneal epithelium in Sjögren’s syndrome. Additionally, an investigation involving small-incision femtosecond laser-assisted intracorneal concave lenticule implantation for keratoconus patients unveiled undisclosed identity dendritic cells within the subepithelial region at the one-month mark post-operation, with their absence observed three months after surgery [22].

In addition, dendritic cells of an undisclosed identity were found in the subepithelial region in keratoconus corneas treated by small-incision femtosecond laser-assisted intracorneal concave lenticule implantation at one month post-operation. No dendritic or inflammatory cells were observed three months after surgery [23].

Considering these observations, this study delves into the diverse types of infiltrated cells present within the stroma of mesenchymal cell therapy for advanced keratoconus through in vivo confocal microscopy (IVCM). Furthermore, we aim to assess disparities between treatments and the post-transplant timeline.

## Patients and methods

### Study approval, design, and subjects

The study encompassed a prospective interventional randomized design, forming a consecutive series of non-masked cases. This collaborative effort involved the Research, Development, and Innovation Department of Vissum Instituto Oftalmologico de Alicante (Grupo Miranza), Miguel Hernandez University (Spain), Optica General (Lebanon), Laser Vision Center (Lebanon), and REVIVA Research and Application Center at Middle East Hospital (Lebanon). The Institutional Review Board (IRB) Ethical Committee of REVIVA granted prospective approval for the study, which adhered to the principles of the Declaration of Helsinki. Informed written consent was obtained from all participating patients for the procedures outlined. The study was officially registered with ClinicalTrials.gov (Code: NCT02932852).

A total of fourteen patients were enrolled and subsequently allocated randomly to three groups: Group 1 (G-1) consisted of patients who received implantation of ADASCs (*n* = 5); Group 2 (G-2) received human dCL, each 120 µm thick (*n* = 5); Group 3 (G-3) received human ADASCs-rCL (*n* = 4). The distribution of laminas in G-2 and G-3 occurred randomly after the decellularization procedure.

Follow-up appointments occurred first at 1 week post-operation, and then at 1, 3, 6, and 12 months post-operation. Thirteen patients successfully completed the full one-year clinical follow-up. Only one patient from G-1 discontinued participation after the first month post-operation, citing personal reasons unrelated to the study. The results of interim analyses conducted at 6 and 12 months have already been published [7–11].

### Patient selection criteria

The study’s inclusion criteria encompassed patients with advanced keratoconus, defined as stage ≥ IV based on the RETICS keratoconus classification [24], who were already designated as candidates for corneal transplantation due to disease severity and associated comorbidities. Participants needed to be at least 18 years of age and exhibit negative serology for human immunodeficiency virus (HIV), hepatitis B (HBV), and hepatitis C (HCV). Conversely, patients with corrected distance visual acuity (CDVA) < 0.1 in the contralateral eye, a history of previous corneal hydrops or central corneal scars, active concomitant inflammatory eye disease, other sight-threatening ocular comorbidities, prior corneal surgical interventions, including collagen cross-linking, pregnancy or breastfeeding status, or a history of systemic malignancy, were excluded from the study.

### Isolation, characterization, and culture of autologous ADASCs

Standard liposuction was conducted on each patient, yielding approximately 250 ml of fat mixed with local anesthesia. The adipose tissue was processed in accordance with previously published protocols [2, 4, 25–27], where autologous ADASCs were isolated, cultured, and characterized by flow cytometry analysis as per the recommendations of the International Federation of Adipose Therapeutics (IFATS). Cell quiescence was induced by reducing the serum concentration to 0.5% for 60 to 80 h prior to transplant, while the absence of apoptosis and aneuploidy was ensured through propidium iodide labeling (Invitrogen, USA) and cell cycle flow cytometry analysis. In G-1, a total of 3 × 10^6^ cells were suspended in 1 ml of phosphate-buffered saline (PBS) and subsequently transplanted intrastromal. In G-3, ADASCs were cultured on decellularized corneal lamina (dCL) of the stroma for 24 h [7, 9] with a seeding density of 0.5 × 10^6^ cells per 1 ml of PBS (12 h on each surface of the lamina).

### Laminas

Human donor corneal stroma characterized by negative viral serology and suitability for human complete corneal transplant were provided by the “Banco de Ojos para el tratamiento de la Ceguera, Centro de Oftalmología Barraquer” in Barcelona (Spain), according to the regulatory directives 2004/23/EC and 206/17/EC established in Spain.

The donor corneas were cut in laminas using a femtosecond laser with a diameter of 9.0 mm and a thickness of 120 µm. The corneal laminas (CL) were subsequently decellularized as before [4]. The efficacy of the decellularization procedure was verified using three distinct approaches: Biochemical digestion in proteinase K, DNA extraction and quantification carried out using a Picogreen Assay kit, and histological nuclear DAPI staining and fluorescence microscopy, alongside hematoxylin and eosin staining [4, 28].

### Surgical procedure

#### Autologous ADASCs implantation

The technique for implanting mesenchymal stem cells has been outlined in preceding publications [7]. Briefly, a 60-kHz IntraLase iFS femtosecond laser (AMO Inc, Irvine, CA) was employed to create a recipient corneal lamellar dissection using a single-pass approach. This generated an intrastromal laminar pocket with a diameter of 9.5 mm, situated at a medium depth corresponding to the thinnest preoperative pachymetry point as measured by anterior segment optical coherence tomography (AS-OCT) (Carl Zeiss, Germany). The anterior side-cut incision was executed at 30°, spanning a 3 mm arc length incision. Parameters mirroring those used in LASIK surgery were employed in the femtosecond laser process. Following this, the intrastromal pocket was opened via blunt dissection, employing a Morlet lamellar dissector (Duckworth & Kent, England). Subsequently, 3 million ADASCs suspended in 1ml PBS were injected into the pocket using a 25-G cannula. Before the cell injection, a 1 mm corneal paracentesis was applied to reduce intraocular pressure and allow a greater volume to be injected into the stromal pocket, while no more than 10% to 30% of the injected volume was estimated to remain within the corneal stroma. Notably, no patients underwent corneal suturing as part of this procedure [7, 9]. A topical antibiotic and steroids (Tobradex; Alcon) were applied at the end of the surgery and were applied every 6 h for 1 week, followed by a descending dose of topical dexamethasone 0.1% (Maxidex, Alcon) for 3 more weeks [7].

#### Lenticule implantation

The same 60-kHz IntraLase iFS femtosecond laser in single-pass mode was utilized in this aspect. A 50° anterior cut assisted with corneal dissection, and the arc length incision measured 4 mm. The intrastromal pocket was opened using blunt dissection by a Morlet lamellar dissector (Duckworth & Kent, England). The lamina was inserted, centered, and gently unfolded through careful tapping and massaging from the epithelial surface of the host cornea. In instances where ADASCs-rCL was utilized (G-3), the pocket was irrigated with a solution containing an additional 1 million autologous ADASCs suspended in 1 ml of PBS, administered via a 25-G cannula. Similar to G-1, a temporal limbal paracentesis was performed just before implantation to reduce the intraocular pressure. Post-insertion, the incision was closed using an interrupted 10/0 nylon suture, which was subsequently removed one week after the surgery. Patients were treated during surgery and post-operatively like ADASCs implantation patients alone [8, 9, 11].

### In vivo confocal microscopy (IVCM) protocol for corneal assessment

IVCM was conducted using a HRT3 RCM (Heidelberg) with a Rostock Cornea Module. This confocal microscope employed a coherent diode laser as its light source, characterized by a wavelength of 670 nm and a minimum resolution of 1024 × 768 at 16-bit [29]. The utilization of coherent light facilitated enhanced image contrast and quality, in particular for visualizing the corneal stroma and its cellular components [30].

For the confocal microscopy assessment, a drop of topical anesthetic (Oxybuprocaine 0.4%) was administered to the eye under evaluation where the confocal microscope was adjusted to + 12 D, and a high-viscosity gel (2.0 mg/g of carbomer) was applied to the front surface of the microscope lens of the Rostock Cornea Module (RCM). A Tomocap was then positioned atop the RCM objective. Patients were guided to maintain a stable position, aligning their gaze with the confocal microscope’s lens and focusing on the light within. The initial focal position was reset to “0” at the superficial epithelial cells of the examined eye. Subsequently, the RCM was rotated either clockwise or anticlockwise within a range of 0 ± 50 μm, and the focal plane was meticulously adjusted to the desired cell layer. To ensure comprehensive visualization, a minimum of four images were captured at intervals of 50 μm in depth, as previously published [29]. These images, integral to the analysis in this study, were obtained at varying stromal depths. However, it’s worth noting that all images were derived from the central diameter region, with a limit of ≤ 9 mm, and situated below the measurement of the intrastromal diameter pocket [7–10, 31].

### ImageJ-based cell characterization and classification

The ImageJ analysis program, developed by the National Institutes of Health, was used. Distinctive morphological features guided the selection of infiltrated cells, setting them apart from host keratocytes characterized by their fusiform shape and specific nuclei traits within different stromal regions [31, 32] Furthermore, infiltrated cells were differentiated from ADASCs that, within the initial 6 months post-surgery, exhibited a unique round shape and distinctive luminosity and refringence compared to regular keratocytes [10, 31].

The meticulous task of cell identification was executed by a panel of four authors (MEZ, MPDM, NM, J.L.A), the inter-concurrence for the first expert was 97%, for the second was 99%, for the third was 92%, and for the fourth was 98%. The intra- concurrence between the four experts was 90%. When all experts agreed on the reproducibility of the ICs identification, each one of the identified ICs underwent independent area measurements by two experts (MEZ and KAJ), being the inter-concurrence 98%, and 96.5% respectively. The intra-concurrence was 92% between the two experts.

**Fig. 1:**
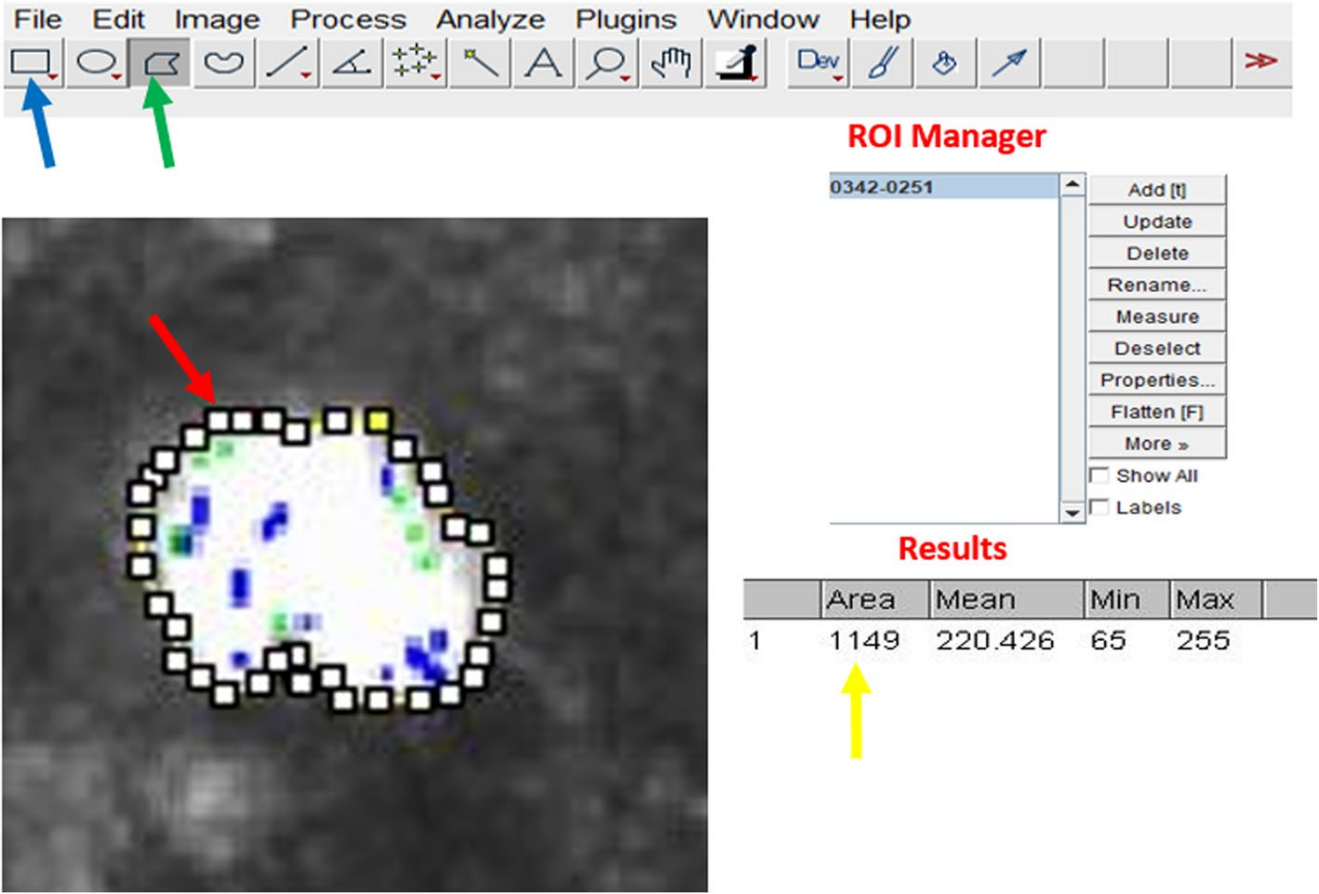
Measurement of the area of the ICs using the Image J software. Open image pressing the icon File (blue arrow), then in the tool bar choose the irregular shape (green arrow), then manually delineate the nuclear periphery of the cell with some squares (red arrow). From the Tool bar, choose Analysis < Tools < Roi Manager and Press Add < Measure, the area measurement is obtained in µm^2^ (yellow arrow)

Within the ImageJ system, the following sequence was employed: Analysis < Tools < Roi Manager (Fig. 1). When calculated area differences exceeded 25% between the two experts, the analysis was reiterated. In categorizing infiltrated cells based on area, the study classified cells as lymphocytes if their area ranged from 50 to 80 µm^2^, granulocytes if within 110 to 220 µm^2^, and monocytes/macrophages for areas exceeding 300 µm^2^. Cells with structures smaller than 50 µm^2^ were designated as cell remnants. In cases falling beyond these size brackets, cell classification was determined by structural attributes and luminance, nuclei, and morphological aspects.

### Statistical analysis and significance evaluation

The statistical evaluations were conducted using Excel 2019, SPSS, and GraphPad Prism 9. Results were presented as mean ± SEM or percentage and assessed for statistical significance using Student’s T-test and F-test, where the latter gauged the variance difference between the two samples. Significance was attributed when both t-test and F-test *P*-values were < 0.05 within the same comparison. Differences among multiple groups were ascertained through a one-way analysis of variance (ANOVA). In all statistical tests, α and *P* values were two-tailed, with the level of significance set at 0.05.

## Results

### Morphological results: GroupWise variations

In the preoperative phase, no infiltrating cells were detected within the tissue samples. However, following surgery, a dynamic shift was observed in infiltrated immune cell composition, spanning from the immediate postoperative period (first month) up to the 12-month mark. Notably, granulocytes emerged as prevalent infiltrates, exhibiting distinct mononuclear and multinuclear forms with varying nuclear morphologies. Neutrophils displayed multi-lobed nuclei, while basophils and eosinophils featured bi-lobed or S-shaped nuclei. Additionally, agranulocytes were identified. Lymphocytes exhibited spherical or indented nuclei, while monocytes/macrophages displayed eccentric or non-eccentric, large indented, or C-shaped nuclei. Furthermore, these infiltrating cell populations exhibited differences in luminosity, cellular structure, and area, reflecting their diverse roles in the intricate processes of immunity and tissue repair (Fig. 1).

#### Distribution of infiltrated cells

In Group 1 (G-1), infiltrated cells were distributed across the anterior, mid, and posterior stromal regions. For Groups 2 (G-2) and 3 (G-3), cells were also found in these same locations, including within the implanted lamina.

#### Shape and luminance of infiltrated cells

**Fig. 2:**
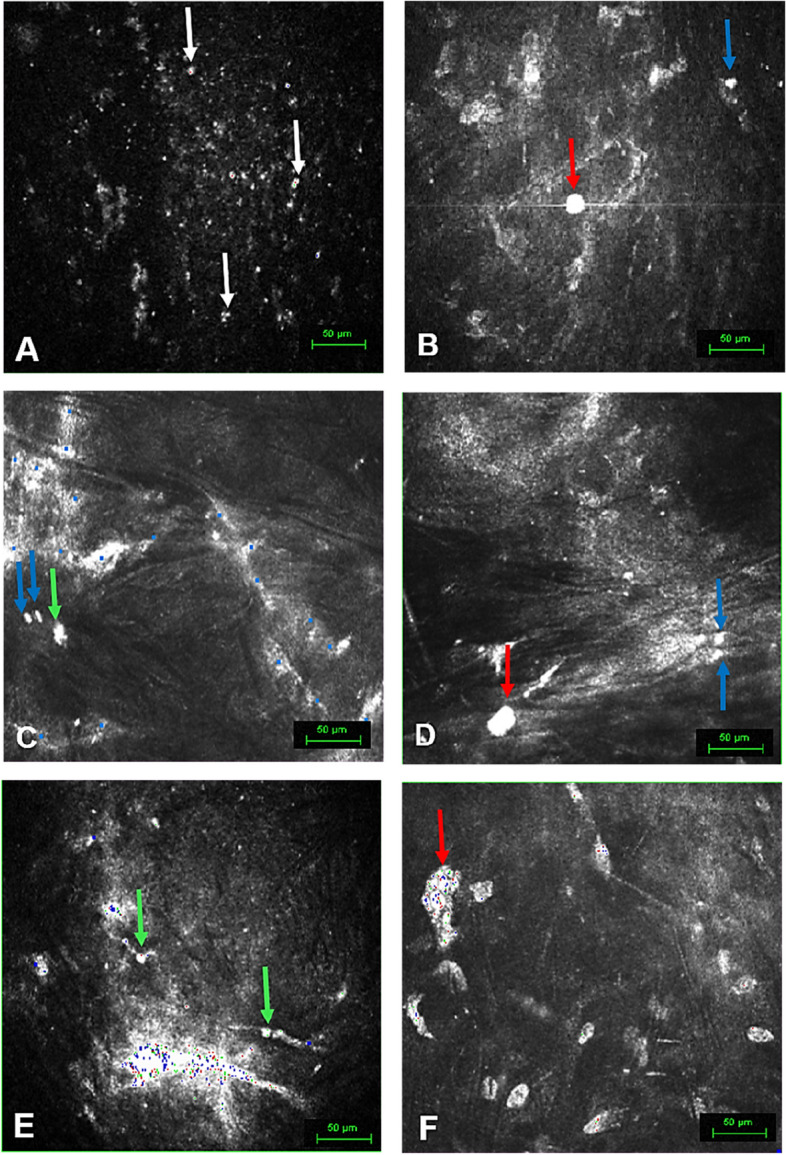
Morphological aspects of infiltrated immune cells in stromal cornea by In Vivo Confocal Microscopy (IVCM). Representative images captured via IVCM provide insights into distinct cellular structures observed in different groups and time points. **A** G-1, Case-3 at one month. The mid stroma displays the presence of small roundish punctiform structures (white arrows), representing remnant cells (cell area < 50 µm^2^). **B** G-1, Case-2 at 12 months. The anterior stroma features a roundish cellular structure (blue arrow) with a nucleus area consistent with a Lymphocyte (72 µm^2^), and the presence of a large-sized cell, rounded and highly reflective, resembling an inflammatory cell (red arrow) with a nucleus area corresponding to a Macrophage (431 µm^2^). **C** G-2, Case-5 at 12 months, on the decellularized cornea lamina (dCL). Notable are two lymphocytes (67 µm^2^, 63 µm^2^, blue arrows) and an elongated granulocyte (227 µm^2^, Green arrow). **D** G-2, Case-8 at 12 months, on the dCL evident, are a highly reflective cell (red arrow) resembling a Macrophage (348 µm^2^), and two lymphocytes (69 µm^2^, 62 µm^2^, blue arrows). **E** G-3, Case-11 at six months. The lamina exhibits rounded, small, and highly reflective cells (green arrows), identified as granulocytes, with respective areas of 102 µm^2^ and 109 µm^2^. **F** G-3, Case-10 at 12 months, anterior stroma. Notice the presence of a large-sized, elongated, highly reflective cell (red arrow), encompassing a nuclear area corresponding to a macrophage size (2631 µm^2^, red arrow), indicating the engulfment of nuclei from other cells

In G-1, remnant cells displayed rounded shapes, with a singular oval exception. These cells exhibited medium luminosity (Fig. 2A). Lymphocytes displayed round or oval shapes, with medium, high, or medium-medium–high luminosity (Fig. 2B). Mononuclear rounded or oval granulocytes were observed, with occasional dendritic periphery. Luminosity levels ranged from medium–high to high. Macrophages appeared round with either multi-nuclear morphology and mid-high luminosity, or mono-nuclear with high luminosity (Fig. 2B).

Within G-2, remnant cells displayed rounded shapes, with one instance of an oval cell. A single star-shaped cell exhibited medium luminosity. Lymphocytes showed round or oval shapes (Fig. 2C, D), and one cell had a semi-star shape. Luminosity levels were medium or medium-medium–high (Fig. 2C, D). Granulocytes were mostly rounded, but some were elongated (Fig. 2C). A semi-circular-semi-star shape was seen in one instance. Luminosity varied from medium–high to high, with one cell having medium luminosity. Macrophages displayed diverse shapes, including oval (Fig. 2D), oval-dendritic, rounded, rounded-multinuclear, or multinuclear with undefined forms. Their luminosity was consistently high.

In G-3, remnant cells took on rounded or oval shapes, accompanied by medium luminance. Lymphocytes displayed rounded or oval shapes, with medium, medium–high, or high luminosity. Granulocytes were mostly mononuclear and rounded (Fig. 2E) but occasionally oval. A mix of shapes, such as star, semi-star, elongated, or triangular, was observed. Luminance levels ranged from medium to high. Macrophages showed diverse shapes, like oval, oval-dendritic, rounded, rounded-multinuclear, or multinuclear with undefined forms (Fig. 2F). Their luminosity remained consistently high.

#### Matrix structure of the corneal pocket

**Fig. 3:**
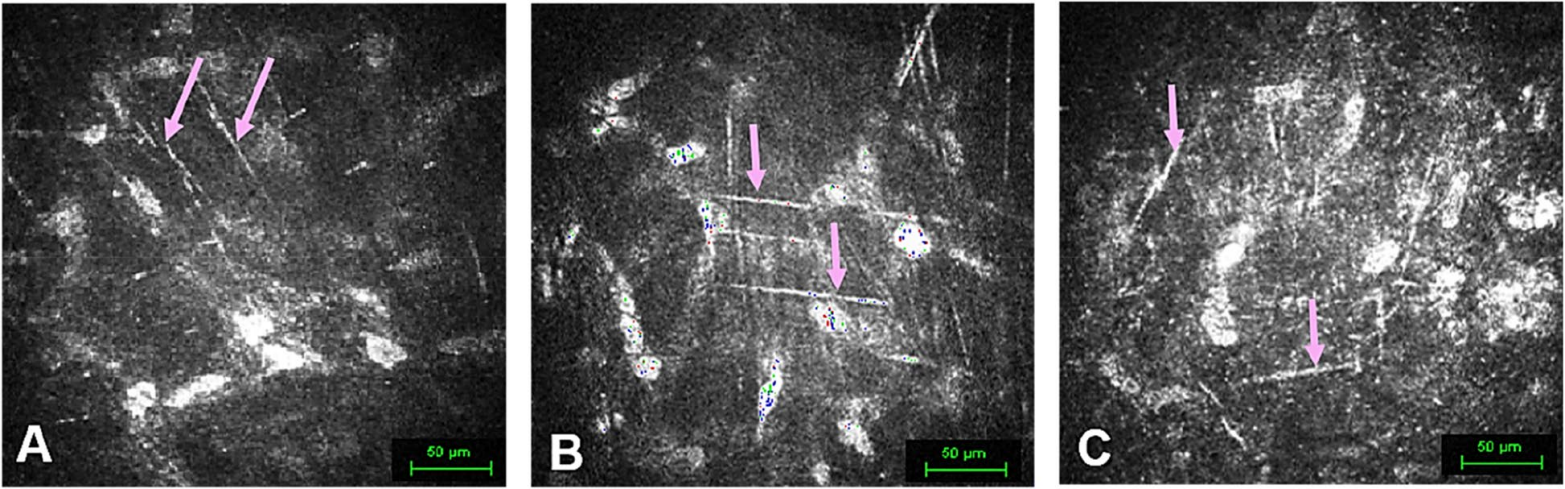
Elongated Needle-Like Structures in Corneal Confocal Microscopy. Representative images illustrate the IVCM observations from different groups and time points of an elongated needle-like structure (NLS). **A** In G-1, Case-2 at 12 months, the corneal pocket matrix presents the presence of elongated NLS (pink arrows). **B** G-2 Case-8 at 6 months on the posterior surface of the dCL. Notice the presence of elongated NLS (pink arrows). **C** In G-3, Case-13 at 1 month, on the posterior surface of the recellularized corneal lamina (rCL), distinct elongated NLS are evident (pink arrows)

All groups exhibited elongated needle-like structures (NLS) within the corneal pocket matrix. The structures displayed varying measurements and were characterized by low to medium luminance. These NLS were observed in G-1, G-2, and G-3 at 1, 3, and up to 6 months, and were observed as more faded at 12 months, revealing no significant variations either among the groups or longitudinally in time (Fig. 3).

### Quantitative results

#### Cell density

**Fig. 4:**
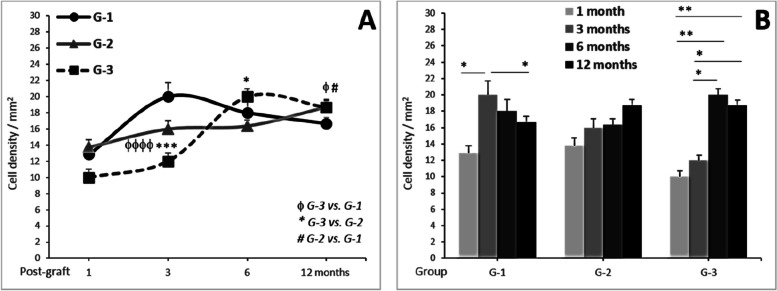
Infiltrated Cell Density in G1, G2, and G3. **A** Comparison between groups (G-1 vs. G-2 vs. G-3) for the same post-transplant time measure. **B** Comparison in function of the post-transplant time (1 vs. 3 vs. 6 vs. 12 months) within the same group. ^**φ**^*P* < .05, ^**φφφ**^*P* < .001, ^**φφφφ**^*P* < .0001: G3 vs. G2 vs. G1. * *P* < .05, ** *P* < .01: 1 vs. 3 vs. 6 vs. 12 months

The extent of stromal infiltration by cells ranged from 1.19% to 6.62%, with an overall mean of 3.23% ± 0.43% across all groups (Fig. 4; Table 1). Notably, a significant increase in cell density was consistently observed in all groups, a trend that was influenced by the specific group rather than the post-operative time (*F* = 10.68, *P* < 0.0001). In fact, the analysis indicated that the increase in cell density was not dependent on the time elapsed after the transplant surgery (*F* = 0.77, *P* = 0.511).

**Table 1:** Significance of the cell density in corneal stroma-infiltrated cells relative to groups

	***Significance between G1 vs. G2 vs. G3 at the indicated time post-transplant (month(s)):***				
	**1**	**3**	**6**	**12**	**Whole time**
F	0.15	8.00	1.57	3.22	10.68
*P*	0.861	< 0.001	0.208	0.040	< 0.0001
	***Significance between 1 vs. 3 vs. 6 vs. 12 months for the indicated group:***				
	**G-1**	**G-2**	**G-3**		**Whole groups**
F	3.25	0.90	0.54		0.77
*P*	0.021	0.440	0.655		0.511

Analyses were performed by One-Way ANOVA

Despite the observed increases in infiltrated cell density within each group over the post-transplant study period, only G-1 (group without lamina) exhibited a significant increase (*F* = 3.25, *P* < 0.05). In contrast, G-2 and G-3 demonstrated progressive increases in cell density from 1 to 12 months post-operation, although these variations did not reach statistical significance over time. The respective cell densities at 1, 3, 6, and 12 months were as follows: G-1 [12.86, 20.00, 18.00, and 16.67 cells/mm^2^], G-2 [13.75, 16.00, 16.36, 18.71 cells/mm^2^], and G-3 [10.00, 12.00, 20.00, and 18.67 cells/mm^2^].

#### Cell size

**Fig. 5:**
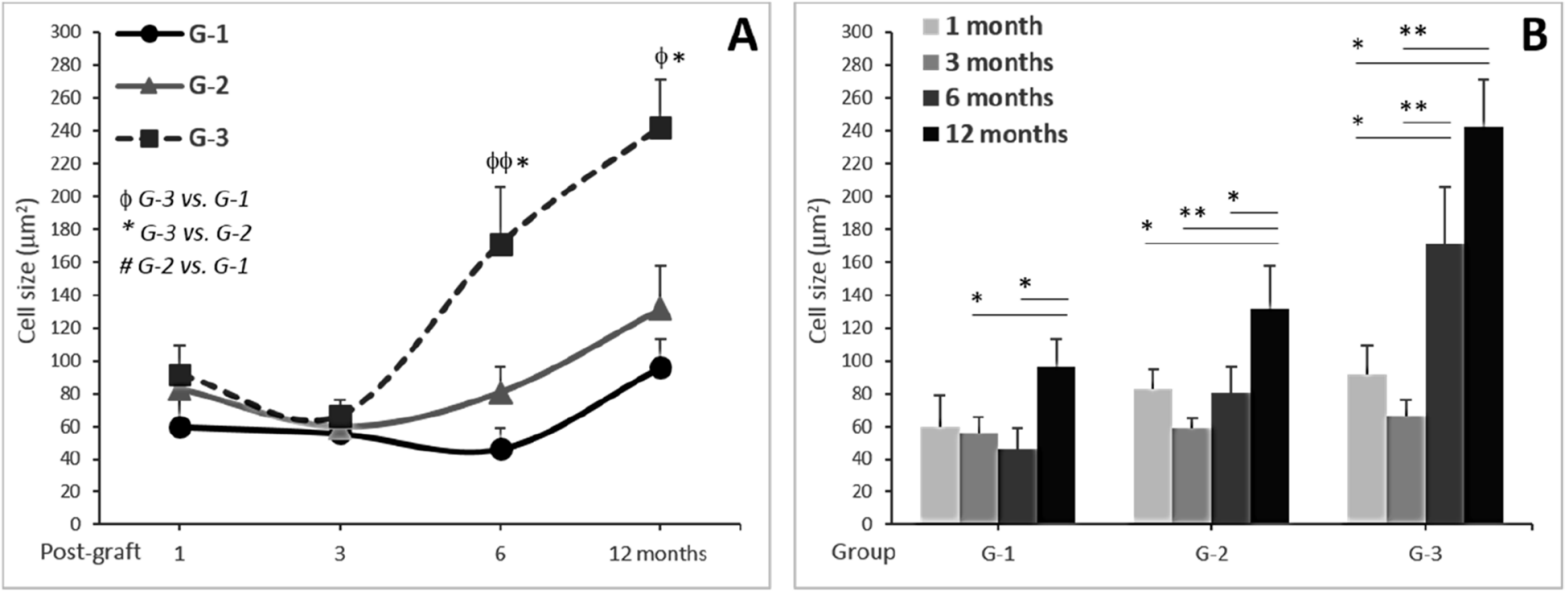
Infiltrated cell size dynamics in different treatment groups and over time. **A** Comparison of cell sizes among G-1, G-2, and G-3 at 1, 3, 6, and 12 months. ^**φ**^*P* < .05, ^**φφ**^*P* < .01: G3 vs. G2 vs. G1. **B** Evolution of cell sizes within each group at 1, 3, 6, and 12 months. *P < 0.05; ** *P* < 0.01

The size of infiltrated cells was initially assessed within each group throughout the post-transplant period of time (Fig. 5). The mean sizes for G-1, G-2, and G-3 were, respectively: at 1 month (59.9 ± 19.4, 82.8 ± 11.6, and 91.4 ± 18.3 µm^2^), at 3 months (55.7 ± 10.3, 59.3 ± 5.8, and 66.5 ± 9.4 µm^2^), at 6 months (46.3 ± 12.6, 80.6 ± 15.8, and 171.2 ± 34.9 µm^2^), and at 12 months (96.1 ± 16.8, 131.7 ± 25.8, and 242.5 ± 28.8 µm^2^)., Larger infiltrated cells were observed predominantly in G-3 more than G-2, in comparison to G-1.

These results demonstrate significant increases in the size of infiltrated cells, primarily attributed to group variances (*F* = 5.76, *P* = 0.003) rather than changes over time (*F* = 2.84, *P* = 0.038) (Table 2). Notably, no significant changes were observed within each group when assessing the size of infiltrated cells separately across different time points. However, significant differences in cell size were evident between groups at 6 and 12 months post-transplant (*F* = 4.68, *P* = 0.02; and *F* = 3.16, *P* = 0.047, respectively).

**Table 2:** Significance of the size variations in corneal stroma-infiltrated cells

	***Significance between G1 vs. G2 vs. G3 at the indicated time post-transplant (month(s)):***				
	**1**	**3**	**6**	**12**	**Whole time**
F	0.67	0.24	4.68	3.16	5.76
*P*	0.520	0.788	0.012	0.047	0.003
	***Significance between 1 vs. 3 vs. 6 vs. 12 months for the indicated group:***				
	**G-1**	**G-2**	**G-3**		**Whole groups**
F	1.95	1.98	1.09		2.84
*P*	0.131	0.120	0.359		0.038

Analyses were performed by One-Way ANOVA

**Fig. 6:**
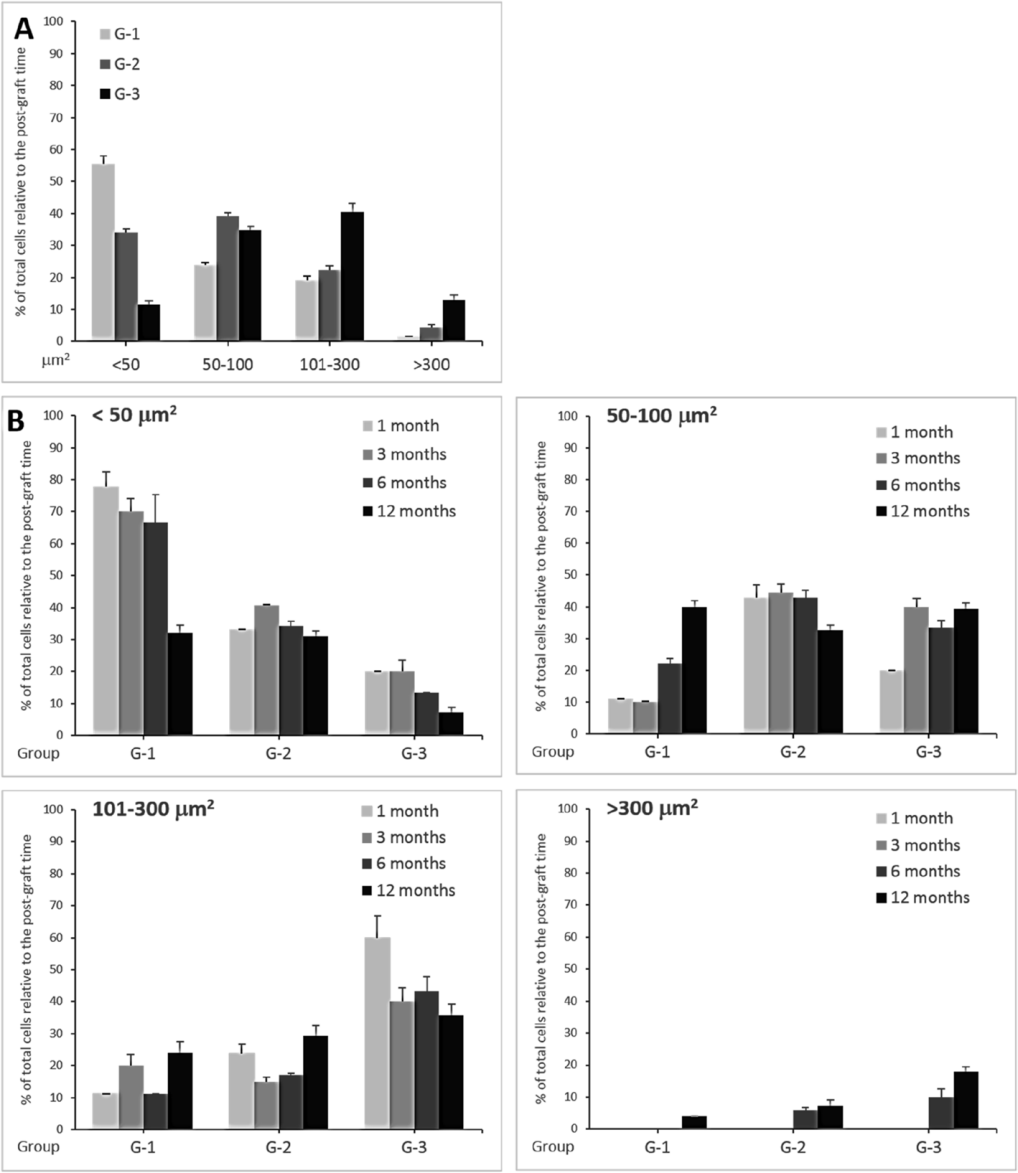
Cell size subgroups analysis. The sizes of infiltrated cells were categorized into 4 subgroups: < 50, 50–100, 101–300, and > 300 µm^2^. **A** grouped results within the same group regardless of post-operative time. **B** distribution among groups (G-1 vs. G-2 vs. G-3) based on the post-operative time. Results are presented as the MEAN ±SEM in the percentage of total cells per each group

To clarify the relationship between groups and the observed variations in cell sizes, we categorized infiltrated cell sizes into four subgroups: < 50, 50–100, 101–300, and > 300 μm^2^. The results are shown in (Fig. 6; Table 3), highlighting the significant increase in cell size primarily observed in G-2 and G-3 (*F* = 110.23, *P* < 0.0001).

**Table 3:** Significance of variations in infiltrated cell size subgroups within corneal stroma

	***Significance between G1 vs. G2 vs. G3 for the indicated size:***				
	** < 50**	**50–100**	**101–300**	** > 300 µm**^**2**^	**Whole sizes**
F	3.16	3.76	0.11	0.40	110.23
*P*	0.047	0.027	0.996	0.678	< .0001
	***Significance between sizes:* < *50 vs. 50–100 vs. 101–300 vs.* > *300 for the indicated group:***				
	**G-1**	**G-2**	**G-3**		**Whole groups**
F	220.9	115.9	21.08		7.80
*P*	< .0001	< .0001	< .0001		0.0005

Analyses were performed by One-Way ANOVA

Regardless of postoperative time, we initially examined the distribution of cell size subgroups across G-1, G-2, and G-3 (Fig. 6A). As shown in Table 3, the percentage of infiltrated cells with sizes < 50 μm^2^ decreases significantly from G-1 to G-2 and G-3 (*F* = 3.16, *P* = 0.047), while increases are evident for other size categories: significantly for 51–100 μm^2^ (*F* = 3.76, *P* = 0.027), but not significantly for 101–300 μm^2^ (*F* = 0.11, *P* = 0.996), and > 300 μm^2^ (*F* = 0.40, *P* = 0.678). These results indicate a pronounced shift towards increased cell size distribution from G-1 to G-3, indicating a lamina-dependent influence (*F* = 7.80.23, *P* = 0.0005).

When analyzing the distribution of infiltrated cell sizes within each subgroup regarding the postoperative time (Fig. 6 upper left), substantial decreases were observed for sizes < 50 μm^2^ in G-1, G-2, and G-3 from 1 to 12 months.

For the subgroup 50–100 μm^2^, the abundance of infiltrated cells was variable among the postoperative months (Fig. 6B upper right). Meanwhile, the percentage of infiltrated cells with sizes 101–300 μm^2^ increased from G-1 to G-3 over the months (Fig. 6B lower left). For the subgroup > 300 μm^2^, these cells only appeared at 12 months in G-1, and at 6 and 12 months in G-2 and G-3 (Fig. 6B down right).

## Discussion

The cornea hosts a diverse population of ICs, including locally resident innate ICs, such as Langerhans cells, mast cells, macrophages, lymphocytes, and innate lymphoid cells [33]. Other ICs, including inflammatory cells, infiltrate the cornea and, especially within the stromal layer, play a pivotal role in modulating the outcomes of cell-based therapies [34].

Given the inherent limitations of conducting biopsies on the grafted cornea, particularly following the transplants of ADASCs, laminas, or combined procedures, and the impossibility of having biopsies longitudinally several times, in order to identify the subsets of infiltrated ICs for their immunophenotyping, our study employs a pioneering approach by utilizing IVCM to explore IC infiltration within the corneal stroma. The use of IVCM provides a remarkable tool [10, 31, 35] for looking into the complex relationship between therapeutic interventions and the host immune response. In fact we have previously used this approach to demonstrate the differentiation of injected MSC into corneal cells [10]. Specifically, we observed a significant increase in corneal keratocyte density in the anterior, mid-, and posterior stroma following the injection of autologous ADASCs in Groups 1 and 3 (G-1). Furthermore, the initially round shape of ADASCs evolved over time, forming clusters and eventually exhibiting an appearance resembling adult corneal stromal cells at 12 months post-surgery, emphasizing the regenerative potential of these treatments.

Nevertheless, the classification of IC subsets, such as the lymphocytes (CD4 + (Th1, Th17, Tregs) and CD8 +), macrophages, NK cells, and others, as well as the measurement of their inflammatory cytokines and angiogenic factors production directly from the grafted cornea, was obviously not possible using our approach and currently poses a significant challenge, without any readily available solutions as of yet.

Some of the results of this report deserve further comment. First, the morphological insights into IC infiltration patterns of this study underscore the pronounced and significant presence of active infiltrated ICs across all therapeutic groups (ADASCs alone in G-1, dCL alone in G-2, and ADASCs-rCL in G-3). Notably, the increase in IC infiltration across all groups suggests that the transplant procedure itself acts as a potential trigger for this immune response. Morphological patterns of ICs within the corneal stroma unveiling shifts in luminosity, structure, area, and cell size enabled their clear distinction from the transplanted ADASCs or differentiated keratocytes [10]. Preoperatively, no infiltrating cells were detected. However, postoperatively, a dynamic change occurred over the year studied. In G-1 and G-2, the predominant infiltrating cells were granulocytes. Conversely, G-3 displayed preeminence in agranulocytes, particularly lymphocytes and macrophages.

The presence of infiltrated ICs within the corneal stroma can yield a range of effects, both positive and negative. The immunobiology of corneal transplantation holds significant importance: even with the use of topical immunosuppression, corneal grafts in recipients with corneal neovascularization or ocular inflammation-related complications, may still face the risk of irreversible rejection, primarily driven by cell-mediated immune responses rather than antibody-mediated reactions [36]. On a positive note, these ICs can contribute to anti-inflammatory responses and tissue repair, in alignment with our findings of increased immune cell density across all treated groups. Our study further reveals that these infiltrated cells actively engage in immune modulation, as evidenced by the absence of adverse clinical effects regardless of the studied group, such as graft rejection, inflammation, immunopathology, infection susceptibility, and vision impairment. This remarkable outcome underscores the efficacy of our cell therapy approach by attenuating potential disadvantages associated with IC infiltration and suggests the transformative potential of our protocol for advanced keratoconus treatment. Our findings also highlight the varying sizes and morphologies of ICs, particularly prominent in G-3 where ADASCs and lamina grafting synergize to impact IC behavior and tissue repair, suggesting their active participation in modulating inflammation and promoting tissue recovery. This phenomenon likely contributes to the positive implications we noted for tissue repair and remodeling. The presence of ICs on the donor corneal endothelium after transplantation [37] and the corneal immune and angiogenic privilege mainly dependent on the heterogenicity of ICs is the key to corneal graft rejection [38], and emphasizes the ongoing crosstalk between immune components and transplanted cells. This dynamic interplay between ICs and transplanted cells aligns with research on corneal antigen-presenting cells [39], immune mechanisms of corneal allograft rejection [40], and the immune privilege of corneal grafts [41].

Using IVCM, NLS has been described in a variety of settings of corneal diseases, including femtosecond laser-assisted keratoplasty [15, 16, 19], generally interpreted as activated keratocytes [42]. In our study these structures were seen in all groups, indicating no direct impact on corneal fibrosis, at least not clinically evident.

Distinct immune cell dynamics were observed across groups:

- The injection of ADASCs performed on G-1 highlighted a notable increase in infiltrated IC density, which persisted for up to 12 months. Cell size also exhibited a significant increase over the year. Remarkably, cell morphology remained relatively stable. The initial increase in IC density from 1 to 3 months, followed by subsequent declines at 6 and 12 months but which remained above the initial level, suggested a changing immune response and tissue repair mechanism, probably due to a mesenchymal-to-keratocytes transition, which reflects an active immunomodulation state immediately after the transplantation, being at an optimum at 3 months and then attenuating. This perspective challenges the conventionally held belief in a linear, time-dependent trajectory of immune response. The observations of ICs on the corneal endothelium of allogeneic corneal transplantation rabbit models [34] and the role of corneal stroma-derived mesenchymal-like stem cells in corneal immunity and wound healing [43] offer insights into the potential therapeutic applications of cell-based approaches.
- In G-2, which received only bioengineered acellular lamina grafting, IC infiltration showed a progressive increase in cell density over the one-year period. Cell morphology showed variations, shifting from rounded shapes to elongated ones, especially at 6 and 12 months: in the context of ICs, an elongated shape could indicate various types of ICs, such as macrophages or dendritic cells, which are known to exhibit elongated or irregular shapes when they are actively involved in surveying and interacting with their microenvironment surroundings. Additionally, infiltrated cell size significantly increased over time, particularly between 6 and 12 months, underscoring the prolonged influence of lamina transplantation on immune cell behavior. In fact, lamellar keratoplasty was reported as an effective and less invasive approach for restoring eye integrity in cases of corneal melting, particularly in inflamed and unstable eyes, allowing for systemic immunosuppression to take effect and the eye to stabilize before considering vision-improving procedures [44].
- In G-3, where the combined approach of ADASCs and acellular lamina grafting was applied, our investigation revealed a distinctive pattern. Similar to the other groups, G-3 showed a progressive increase in cell density over the study period, particularly within the lamina and posterior surface. Notably, G-3 exhibited statistically significant higher IC density than G-1 and G-2. However, what sets this group apart are the multifaceted changes in cell morphology that extend over time. Diverse cell shapes, including elongated, irregular, and dendritic forms, became more noticeable. The larger size of ICs, coupled with the decreased presence of debris and smaller cells (size < 50 µm^2^) within this cell therapy group, could indicate an ongoing immune-mediated remodeling process, suggesting these ICs might contribute to the overall efficacy of the cell therapy. This conversion toward larger cell sizes suggests the implication of macrophages in phagocytosis and extracellular matrix modulation, both of which are pivotal for tissue regeneration. In fact, MSCs modulate the immune response by secreting cytokines, growth factors, and extracellular matrix proteins [45, 46] that inhibit the infiltration of inflammatory cells following injury and promote a healing phenotype via M2 macrophage polarization [47].
- While our study focuses on mesenchymal extraocular cells, parallels can be drawn with the work of Yam and colleagues [48], who investigate intrastromal injection of cultivated human corneal stromal keratocytes,while their emphasis is on corneal opacities, the safety and feasibility aspects of their approach align with our considerations of immune responses and infiltrating cells.
- In CONCLUSION, our investigation into the immune responses in corneal cell therapy offers insights into the complex interactions within the corneal microenvironment and their potential impact on therapeutic outcomes. The prospects of optimizing corneal cell therapies for enhanced clinical outcomes remain promising [49, 50]. Understanding the mechanisms and dynamics of immune cell infiltration into the corneal stroma after ADASCs-rCL grafting is important for developing strategies aimed at improving graft outcomes. Further research is needed to investigate the specific types of immune cells involved in the infiltration process and their role in cornea repair, importantly identifying in vivo inflammatory cells such as Th1/Th17 in the corneal stroma of patients, which represents a crucial avenue for future assessments.

## Abbreviations

ADASCs: Adipose-derived adult stem cell
ADASCs-rCL: ADASCs recellularized onto dCL:
CL: Corneal laminas
dCL: Decellularized corneal Lamina
IFATS: International Federation of Adipose Therapeutics
IVCM: In vivo confocal microscopy
NLS: Needle-like structures
PBS: Phosphate-buffered saline
